# Impaired emotion recognition in *Cntnap2*-deficient mice is associated with hyper-synchronous prefrontal cortex neuronal activity

**DOI:** 10.1038/s41380-024-02754-8

**Published:** 2024-09-17

**Authors:** Alok Nath Mohapatra, Renad Jabarin, Natali Ray, Shai Netser, Shlomo Wagner

**Affiliations:** Sagol Department of Neurobiology, Faculty of Natural Sciences, University of Haifa, Haifa, Israel

**Keywords:** Neuroscience, Physiology

## Abstract

Individuals diagnosed with autism spectrum disorder (ASD) show difficulty in recognizing emotions in others, a process termed emotion recognition. While human fMRI studies linked multiple brain areas to emotion recognition, the specific mechanisms underlying impaired emotion recognition in ASD are not clear. Here, we employed an emotional state preference (ESP) task to show that *Cntnap2*-knockout (KO) mice, an established ASD model, do not distinguish between conspecifics according to their emotional state. We assessed brain-wide local-field potential (LFP) signals during various social behavior tasks and found that *Cntnap2*-KO mice exhibited higher LFP theta and gamma rhythmicity than did C57BL/6J mice, even at rest. Specifically, *Cntnap2*-KO mice showed increased theta coherence, especially between the prelimbic cortex (PrL) and the hypothalamic paraventricular nucleus, during social behavior. Moreover, we observed significantly increased Granger causality of theta rhythmicity between these two brain areas, across several types of social behavior tasks. Finally, optogenetic stimulation of PrL pyramidal neurons in C57BL/6J mice impaired their social discrimination abilities, including in ESP. Together, these results suggest that increased rhythmicity of PrL pyramidal neuronal activity and its hyper-synchronization with specific brain regions are involved in the impaired emotion recognition exhibited by *Cntnap2*-KO mice.

## Introduction

Social cognition involves the perception and interpretation of social cues transmitted between individuals, processes crucial for the appropriate adaptation of a subject to its social environment [1, 2]. The ability to recognize the emotional state of other individuals [3–5], termed emotion recognition [6], is crucial for a wide range of prosocial behaviors, such as emotion contagion, empathy, and helping behavior [7–9] and is known to be impaired in Individuals diagnosed with autism spectrum disorder (ASD) [10–12]. While fMRI studies have supplied considerable information regarding active brain areas during tasks of emotion recognition [4, 13–16], including frontal lobe and amygdalar regions [3, 5], specific deficits in brain activity and neuronal network dynamics that lead to impaired emotion recognition in ASD remain elusive. One valuable tool for deciphering such deficits is animal models of ASD, which allow for invasive monitoring and manipulation of brain neural activity during social behavior [17]. Yet, reliable behavioral tasks to assess emotion recognition in animal models were lacking until only recently.

Of late, we and others have demonstrated that mice can discriminate between conspecifics according to the conspecific’s emotional state, thus providing a tool for assessing emotion recognition in murine models of ASD [18–20]. This observation led to the development of a new behavioral task, termed by us emotional state preference (ESP). This task is based on the ability to discriminate between two stimulus animals simultaneously presented to a subject mouse, one of which was emotionally aroused by a given manipulation. While Ferretti et al. showed that C57BL/6J mice preferred to investigate fearful, stressed and relieved conspecifics more than neutral stimulus animals [18], we demonstrated that C57BL/6J mice preferred to investigate a stimulus animal socially isolated for seven days over a group-housed stimulus animal [19].

We previously demonstrated that mice expressing the A350V-encoding mutation in the *Iqsec2* gene, a mutation associated with ASD in humans [21], showed a specific deficit in the ESP task [19]. Here, we sought to further validate this observation in another ASD murine model, so as to assess the generality of this deficit. One of the most established murine models of ASD is the *Cntanp2*-knockout mouse line, which does not express a functional copy of the *Contactin-associated protein 2* gene [22]. Previous studies showed that such mice exhibit a reduced tendency for social interaction and that this can be reversed by inhibiting the excitability of medial prefrontal cortex (mPFC) pyramidal neurons or by activating inhibitory GABAergic interneurons in this region [23]. Notably, recognition of stress and relieved states by C57BL/6J (C57) mice was found to be dependent upon somatostatin-expressing GABA-ergic inhibitory interneurons in the mPFC [20]. However, emotion recognition by *Cntnap2*-deficient mice has yet to be examined.

Here, we addressed this issue by employing the ESP paradigm to examine emotion recognition in *Cntnap2*^−/−^ (KO) mice. We found that these mice do not prefer to investigate aroused over neutral conspecifics. Surprisingly, even wild-type (WT) offspring of *Cntnap2*^−/+^ mice were impaired in this behavior, suggesting that the etiology of this impairment involves not only a subject’s genotype, but also the genotype of its parents and/or littermates. We further simultaneously recorded local field potential (LFP) signals from multiple brain areas linked to social behavior and found hyperactive theta and gamma rhythmicity in the brains of KO mice, as compared to C57 mice. Moreover, synchronization between the prelimbic (PrL) area of the mPFC and several hypothalamic and amygdalar areas was consistently modified in KO mice across multiple social discrimination tasks. Finally, using optogenetic stimulation, we demonstrated that stimulating PrL pyramidal neurons at either theta or gamma frequency impaired the ability of C57 mice to discriminate between various types of conspecifics and specifically, between emotionally-aroused and neutral stimuli, similarly to KO mice. These results suggest that the modified synchronization of PrL neural activity exhibited by KO mice does not merely cause social avoidance, as previously suggested [24, 25], but rather interferes with social recognition and discrimination, thus creating a complex deficit which seems to be highly related to ASD.

## Results

### *Cntnap2* KO and WT littermates exhibit impaired emotion recognition

**Fig. 1:**
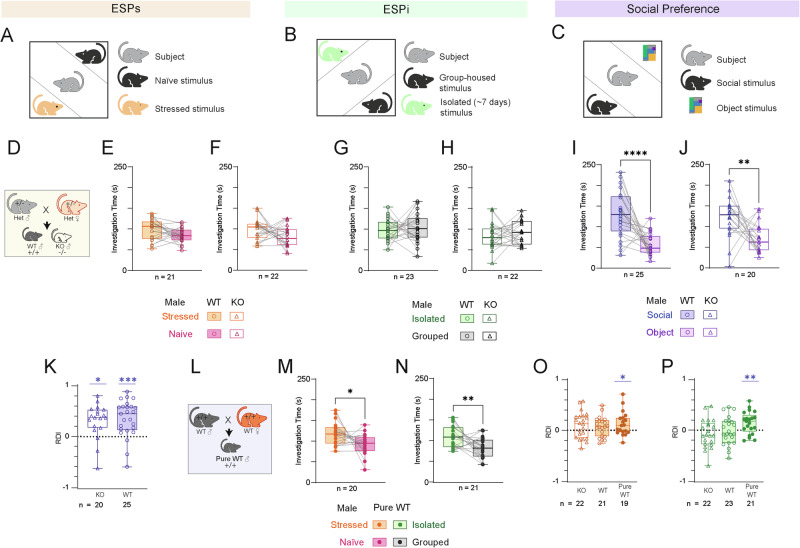
*Cntnap2*-KO and WT littermates exhibit impaired emotional state preference (ESP) behavior. **A** Schematic representation of the ESPs task. **B** As in (**A**), for the ESPi task. **C** As in (**A**), for the SP task. **D** The breeding scheme of WT and KO littermates. **E** Median (in box plot^) time dedicated by WT littermates to investigating the stressed (orange) stimulus or the naïve (pink) stimulus during the 5 min encounter period of the ESPs task (paired t-test, *n* = 20 sessions, t_20_ = 1.869, *p* = 0.0763). **F** As in (**E**), for KO littermates (Paired t-test, t_21_ = 1.870, *p* = 0.0755). **G** As in (**E**), for ESPi (t_22_ = 0.5879, *p* = 0.5879). **H** As in (**F**), for ESPi (t_21_ = 0.8667, *p* = 0.3959). **I** As in (**E**), for SP (t_24_ = 4.934, *p* < 0.0001). **J** As in (**F**), for SP (t_19_ = 4.934, *p* = 0.0066). **K** Relative discrimination index (RDI) for KO (One sample t-test, t_13_ = 2.616, *p* < 0.05) and WT (t_24_ = 4.978, *p* < 0.001) littermates. **L** The breeding scheme of pure WT produced by breeding two WT littermates. **M** As in (**E**), for pure WT animals in the ESPs task (t_18_ = 2.403, *p* = 0.0273). **N** As in (**M**), for the ESPi task (t_20_ = 2.987, *p* = 0.0073). **O** As in (**K**), for the ESPs task (KO, t_21_ = 1.868, *p* > 0.05; WT, (t_20_ = 1.603, *p* > 0.05; Pure WT, t_18_ = 2.397, *p* < 0.05)). **P** As in (**K**), for the ESPi task (KO, t_21_ = −1.003, *p* > 0.05); WT, t_22_ = −0.276, *p* > 0.05; pure WT, t_20_ = 3.073, *p* < 0.01). ^Box plot represents 25 to 75 percentiles of the distribution, while the bold line is the median of the distribution. Whiskers represent the smallest and largest values in the distribution. *p < 0.05, **p < 0.01, ****p < 0.0001, One Sample or Paired t-test.

Since patients diagnosed with ASD are known to exhibit impaired emotion recognition [10–12], we first examined whether KO mice are impaired in terms of ESP using two variations of the test that assesses this trait. Specifically, in the stress version of the ESP task (ESPs), subjects were simultaneously exposed to a stressed stimulus animal and a naive animal (Fig. 1A), whereas in the isolation version of ESP (ESPi), subjects encountered a socially-isolated stimulus animal and a group-housed animal (Fig. 1B). The stimulus animals were separately placed in triangular chambers, located in opposite corners of the arena, which enabled restricted interaction between the subject and the stimulus animal via a metal mesh [26]. As a control, we conducted a social preference (SP) task in which the subjects encountered a novel same-sex animal stimulus and an object (Fig. 1C) [27]. Surprisingly, we found that both WT and KO male offspring of *Cntnap2*^+/−^ parents (Fig. 1D), did not discriminate between the two stimulus animals in either of the ESPs or ESPi tasks (Fig. 1E–H). In contrast, both WT and KO mice showed normal behavior in the SP task, investigating the stimulus animal for significantly more time than the object (Fig. 1I, J). Accordingly, both groups exhibited a relative discrimination index (RDI) significantly higher than zero in the SP test (Fig. 1K). Thus, KO mice and their WT littermates exhibited similar impairments, specifically in emotion recognition. Since previous studies showed that behavioral deficits in genetic animal models of ASD are sometimes exhibited by WT offspring of mutant parents [28, 29], we generated “pure WT” mice by breeding two WT parents [30] (Fig. 1L) and found that these animals perform normally in the ESPs and ESPi tasks (Fig. 1M, N). Accordingly, only the pure WT animals exhibited RDI values which were significantly higher than zero in both ESPs and ESPi, while KO and WT animals did not show that in any of the two versions of ESP (Fig. 1O, P). Altogether, these results demonstrate that *Cntnap2*-KO mice are impaired in terms of their ESP behavior, although the etiology of this impairment involves not only the subject’s genotype but also the genotypes of its parents and/or littermates. Since the genetic background of the KO mice is that of the C57 mouse strain, we continued this study by comparing brain activity of KO and C57 mice during social behavior.

To that end, we employed four behavioral tasks, namely, the SP and the ESPi tasks, a sex preference (SxP) task in which the subjects encountered a male and a female age-matched stimulus animal, and a free social interaction (FSI) task involving a same-sex, age-matched novel stimulus animal. For these experiments, we only used male subjects, as both C57 and pure WT female mice did not show a preference in the ESPi task, which seems to be sex-specific (Fig. S1A–E). An electrode array was implanted in the brain of each subject as previously described [31] and behavioral experiments were conducted 3 days later. Each of the recorded subjects (11 C57 and 15 KO mice) performed up to three sessions of each task (see timeline in Fig. S2A), and the results from each session were separately analyzed.

**Fig. 2:**
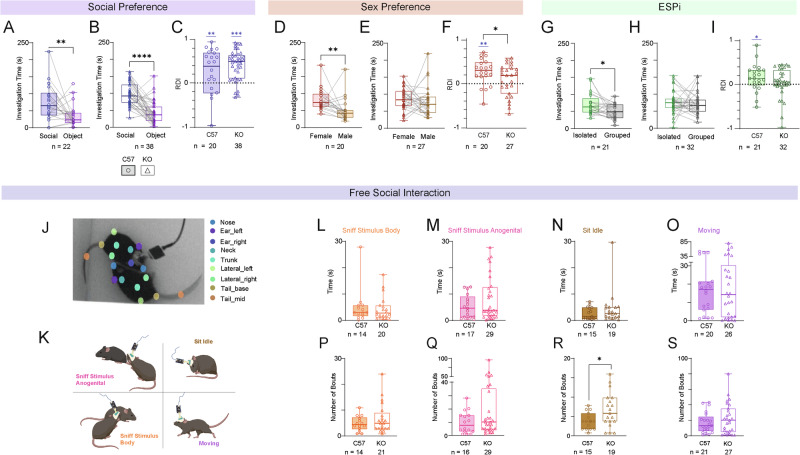
*Cntnap2*-KO male mice exhibit specific deficits in the ESPi and SxP tasks. **A** Median time dedicated by C57 mice to investigate the animal (blue) or object (purple) stimulus during the 5 min encounter period of the SP task (Wilcoxon matched-pairs signed rank test, W = −161, *p* = 0.0074). **B** As in (**B**), for KO mice (W = -639, *p* < 0.0001). **C** Relative discrimination index (RDI) for C57 and KO mice (One sample t-test- C57BL\6J: t_19_ = 2.853, *p* = 0.01, KO: t_37_ = 7.779, *p* = 0.000; Wilcoxon matched-pairs signed rank test- W = 614, *p* = 0.382). **D** As in (**A**), for the SxP task (W = −146, *p* = 0.0049). **E** As in (**B**), for the SxP task (W = −80, *p* = 0.3483). **F** As in (**C**), for the SxP task (One sample t-test- C57: t_19_ = 3.925, *p* = 0.001; Unpaired t-test, t_45_ = 2.013, *p* < 0.05). **G** As in (**A**), for the ESPi task (W = −121, *p* = 0.0351). **H** As in (**B**), for the ESPi task (W = −152, *p* = 0.16). **I** As in (**C**), for the ESPi task (One sample t-test- C57: t_20_ = 2.227, *p* = 0.038, Wilcoxon matched-pairs signed rank test- W = 812, *p* = 0.344). **J** Snapshot of a DeepLabCut tracking of a tethered subject interacting with a stimulus animal during a FSI session. Notice the body parts marked by the trained DeepLabCut model and corresponding names of each body part denoted in the right colored labels. **K** Schematic representations of the four poses analyzed using DeepLabCut + SimBA analyses. **L** Median time dedicated by C57 (left bar) and KO (right bar) mice to sniff the body of the stimulus animal during the 5 min encounter period of the FSI task (Mann Whitney test, U = 128, *p* = 0.6913). **M** As in (**O**), for sniffing the anogenital region of the stimulus animal (U = 211, *p* = 0.4297). **N** As in (**O**), for sitting idle (U = 112, *p* = 0.3024). **O** As in (**O**), for moving (U = 250, *p* = 0.8349). **P** As in (**L**) for the number of sniff the body of the stimulus animal bouts (14 C57 and 21 KO sessions annotated with this event; Mann Whitney test, U = 129, *p* = 0.5529). **Q** As in (**M**) for the number of sniffing the anogenital region of the stimulus animal bouts (19 C57 and 29 KO; U = 182.5, *p* = 0.245). **R** As in (**N**) for the number of sitting idle bouts (15 C57 and 19 KO; Unpaired t test, t_32_ = 2.191, *p* = 0.0358). **S** As in (**O**) for the number of moving bouts (21 C57 and 27 KO; U = 257.5, *p* = 0.5953). *p < 0.05, **p < 0.01, ****p < 0.0001, Mann-Whitney test or Unpaired t-test. See Fig. S2 for more details regarding the SimBA model.

We found that both genotypes (C57 and KO) exhibited a significant preference to investigate the social stimulus over the object in the SP task (Fig. 2A, B), with no difference in RDI between them (Fig. 2C). However, while C57 mice showed a preference to investigate the female stimulus animal in the SxP task (Fig. 2D), KO mice did not show such a preference (Fig. 2E) and the preference index differed significantly between the two groups (Fig. 2F). As for ESPi, C57 mice exhibited a significant preference, in contrast to KO mice (Fig. 2G, H). However, this preference was weak, such that no significant difference in RDI was observed between the two groups (Fig. 2I).

In the last task (i.e., the FSI task), each subject was exposed to a novel stimulus animal for 5 min in the empty arena. The behavior of the subject was tracked using DeepLabCut (DLC) software [32], and the time and number of four behavioral types were quantified by SimBA behavioral segmentation analysis [33] (Fig. 2J, K and Fig. S3B–E). We found no differences between the two genotypes in any of the behavioral variables analyzed (Fig. 2L–S), other than the number of “sitting idle” events (Fig. 2R). Overall, we identified deficits in the social behavior of KO mice, specifically in the ESPi and SxP tasks.

### *Cntnap2*-KO mice display augmented LFP theta and gamma rhythmicity

**Fig. 3:**
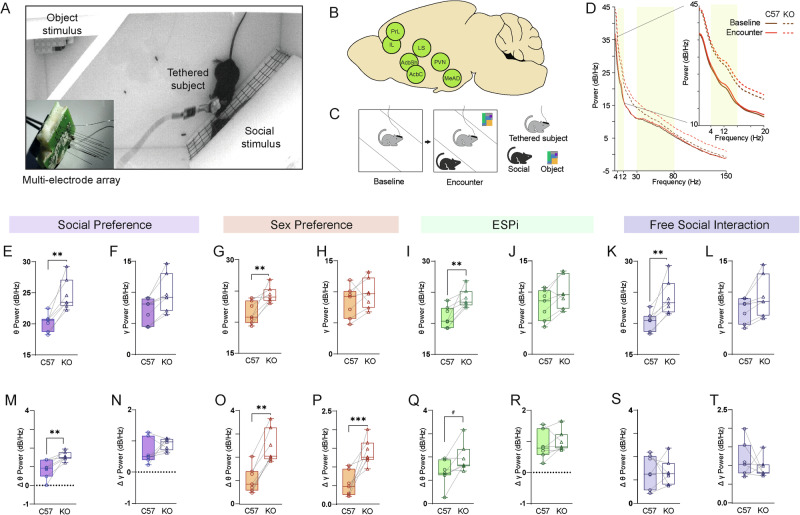
*Cntnap2*-KO mice exhibit higher levels of theta and gamma power in all tasks. **A** A picture of a recorded subject mouse in the arena during the SP task. Inset—a picture of an electrode array. **B** Schematic representation of the seven recorded brain areas analyzed. **C** Schematic representation of the two stages of a recording session (SP, in this example), i.e., baseline and encounter. **D** Example plotted power spectral density (PSD) profiles for LFP signals recorded from C57 (continuous lines) and KO (dashed lines) subject mice during the baseline (purple) and encounter (red) periods of a single SP session. Yellow areas denote the theta (4–12 Hz) and gamma (30–80 Hz) bands. Inset—the same curves showing the theta range at higher resolution. **E** Median theta power of LFP signals recorded during the baseline period of all SP experiments, across all seven brain regions, for C57 (left bar) and KO (right bar) subjects (Unpaired t test, *n* = 7 Brain regions, t_12_ = 4.036, *p* = 0.0016). **F** As in (**E**), for gamma power (t_12_ = 2.053, *p* = 0.0626). As in (**E**, **F**), for SxP session (**G**): t_12_ = 3.457, *p* = 0.0047; (**H**): t_12_ = 0.9996, *p* = 0.3372). As in (**E**, **F**), for ESPi sessions (**I**): t_12_ = 3.476, *p* = 0.0046; (**J**): t_12_ = 1.437, *p* = 0.176). As in (**E**, **F**), for FSI session (**K**): t_12_ = 3.204, *p* = 0.0076; (**L**): t_12_ = 1.587, *p* = 0.1385). **M**–**T** As in (**E**–**L**), for Δpower during the encounter, as compared to baseline. **M**: t_12_ = 3.48, *p* = 0.0045; (**N**): t_12_ = 1.437, *p* = 0.1726. **O** t_12_ = 4.101, *p* = 0.0015; **P**: t_12_ = 4.704, *p* = 0.0005; (**Q**): t_12_ = 2.024, *p* = 0.0658; (**R**): t_12_ = 0.59, *p* = 0.56; (**S**): t_12_ = 0.081, *p* = 0.9363; (**T**): t_12_ = 0.9117, *p* = 0.3799. #*p* < 0.066, *p < 0.05, **p < 0.01, ****p < 0.0001, Unpaired t-test.

To assess brain activity during social behavior, we analyzed local field potential (LFP) signals recorded from electrode array-implanted mice while they performed the behavioral tasks described above (Fig. 3A). The location of each electrode tip was verified post-mortem in each mouse (Fig. S2F) and only regions where an adequate sample size (*n* ≥ 5 sessions) was available for both C57 and KO mice were considered. Of all recorded brain areas (nine common to C57 and KO mice), we analyzed signals from seven social behavior-associated brain areas (see Methods, Table S1). The analyzed brain areas included the anterodorsal part of the medial amygdala (MeAD), the nucleus accumbens core (AcbC) and shell (AcbSh), the prelimbic (PrL) and infralimbic (IL) cortices, the hypothalamic paraventricular nucleus (PVN) and the lateral septum (LS) (Fig. 3B).

We first analyzed the mean power of theta and gamma rhythmicity of LFP signals recorded along a 5 min-long baseline period, during which time there were no stimuli in the arena, and averaged it across all the brain regions listed above (Fig. 3C, D). We measured higher baseline mean theta power in KO mice, as compared to C57 mice, with this difference being statistically significant in all four tasks (Fig. 3E, G, I, K). Baseline gamma power showed a similar tendency, albeit without statistical significance (Fig. 3F, H, J, L). These differences between the genotypes were observed even when we considered only the first session conducted by each mouse, thus excluding the possibility that the changes were associated with expectation of a social encounter (Fig. S2G–H). These results suggest that KO mice exhibited higher level of theta power, even before the beginning of the social encounter.

We next analyzed the mean change (Δ) in theta and gamma powers recorded during the 5 min-long encounter, as compared to baseline power values (Fig. 3D). In all cases, LFP power during the encounter was higher than at baseline, as reflected by positive Δpower values in both the theta and gamma bands (Fig. 3M–T). However, the KO mice exhibited significantly (or trendily in the ESPi case) higher increases in theta power, compared to C57 mice, in all three social discrimination tasks (Fig. 3M, O, Q), while the KO gamma Δpower was significantly higher only in the SxP task (Fig. 3N, P, R). It should be noted that while during the SP and ESPi tasks the difference in theta power change was most prominent during the first minute of the encounter, in the SxP task it was kept constant throughout the encounter (Figs S3A–C, E–G). In contrast to the discrimination tasks, we did not find a significant difference in Δpower between genotypes during the FSI task (Fig. 3S, T), not even during the first minute of encounter (Fig. S4D, H). Assuming that LFP theta rhythmicity is enhanced by internal states such as attention and arousal [34–38], these results suggest an initially higher internal state in KO mice compared to C57 mice, which was further enhanced during the early encounter stage of the various social discrimination tasks.

### *Cntnap2*-KO mice exhibit hyper-synchronous brain activity during social interaction

**Fig. 4:**
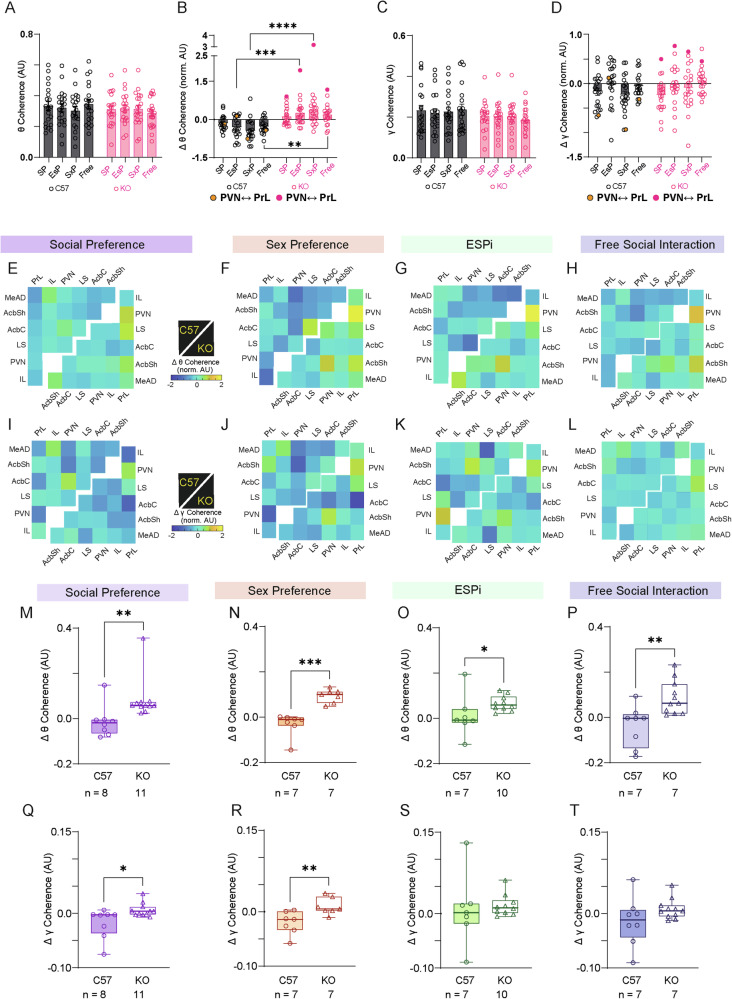
KO mice show higher theta coherence during the various tasks, especially between the PrL and PVN. **A** Mean (±SEM) theta coherence across all brain region pairs (*n* = 20 for each task) during the baseline of all tasks in C57 (black bars) and KO (red bars) subjects (Two-way ANOVA. Genotype: F_1, 152_ = 1.459, P = 0.3104; Tasks: F_3, 152_ = 0.1049, P = 0.9571; Interaction: F_3, 152_ = 0.6683, P = 0.5727). **B** Mean (±SEM) normalized change in theta coherence across all pairs of brain regions (*n* = 20 for each task) during the encounter period, as compared to baseline, of all tasks conducted by C57 (black bars) and KO (red bars) subjects. The data points representing the coherence between the PrL and PVN are denoted as filled dots. (2-way ANOVA. Genotype: F_1, 152_ = 52.27, P < 0.0001; Tasks: F_3, 152_ = 0.04024, P = 0.9892; Interaction: F_3, 152_ = 2.496, P = 0.062). **C**, **D** As in (**A**, **B**), for gamma coherence. **C**: Genotype: F_1, 152_ = 4.081, P = 0.0451; Tasks: F_3, 152_ = 0.061, P = 0.98; Interaction: F_3, 152_ = 0.3091, P = 0.8188). **D**: Genotype: F_1, 152_ = 0.4696, P = 0.4942; Tasks: F_3, 152_ = 3.260, P = 0.0232; Interaction: F_3, 152_ = 0.9164, P = 0.4346. **E** Heat-map matrices of the normalized change in theta coherence during the SP task across all recorded pairs of brain regions for C57 (upper right matrix) and KO mice (lower left matrix). Note that white spots represent a brain region pairs with an inadequate sample size. **F** As in (**E**), for SxP sessions. **G** As in (**E**), for ESPi sessions. **H** As in (**E**), for FSI sessions. **I**–**L** As in (**E**–**H**), for the normalized change in gamma coherence. **M** Median change in theta coherence between the PrL and PVN across all SP sessions for the C57 (circles, left) and KO (triangles, right) subjects (Mann Whitney test, U = 10, P = 0.0036). **N** As in (**M**), for SxP sessions (U = 0, P = 0.0006). **O** As in (**M**), for ESPi sessions (U = 12, P = 0.025). **P** As in (**M**), for FSI session (U = 10, P = 0.0036). **Q**–**T** As in (**M**–**P**), for the change in gamma coherence. **Q**: U = 14, P = 0.0121; (**R**)**:** SxP: U = 4, P = 0.007; (**S**)**:** ISP: U = 26, P = 0.4173; (**T**): FSI: U = 4, P = 0.1518. **A**–**D**: **p < 0.01, ***p < 0.001, ****p < 0.0001 Šídák’s multiple comparison test after two-way ANOVA; (**M**–**T**)**:** *p < 0.05, **p < 0.01, ***p < 0.001, Mann Whitney test.

While the power of LFP rhythmicity may reflect the internal state of the animals [39, 40], LFP coherence between brain regions is thought to reflect their functional connectivity [41]. When analyzing the coherence of LFP rhythmicity across all pairs of brain regions considered, we found that, unlike mean power, the mean coherence did not differ between tasks and genotypes during the baseline period for both theta and gamma rhythms (Fig. 4A, C). These results suggest that the mean coherence was resilient to the initial internal state that affected the mean LFP power (Fig. 3E–L). However, the normalized change in theta coherence during the encounter showed a significant difference (after correcting for multiple comparisons) between genotypes, with KO animals showing higher coherence changes in all tasks, other than the SP task (Fig. 4B). No significant differences were observed for the change in gamma coherence (Fig. 4D). Thus, theta rhythmicity during the encounter stage of the various tasks seems to be hyper-synchronous across the recorded brain regions in KO mice. Notably, the coherence between the PrL and PVN was especially high in KO animals, as compared to C57 mice, for both theta and gamma rhythmicity (Fig. 4B, D, filled circles; see also heat-maps in Fig. 4E–L). We, therefore, analyzed the coherence between this pair of brain regions in each task separately and found a significantly higher encounter-induced change in theta coherence in KO mice across all tasks (Fig. 4M–P). At the same time, changes in gamma coherence showed a significant difference only for the SP and SxP tasks (Fig. 4Q, S). Overall, these results demonstrate hyper-synchronization of LFP theta rhythmicity in KO mice during social behavior, especially between the PrL and PVN.

### The prelimbic cortex in *Cntnap2*-KO mice shows modified synchronization with other regions during social interaction

**Fig. 5:**
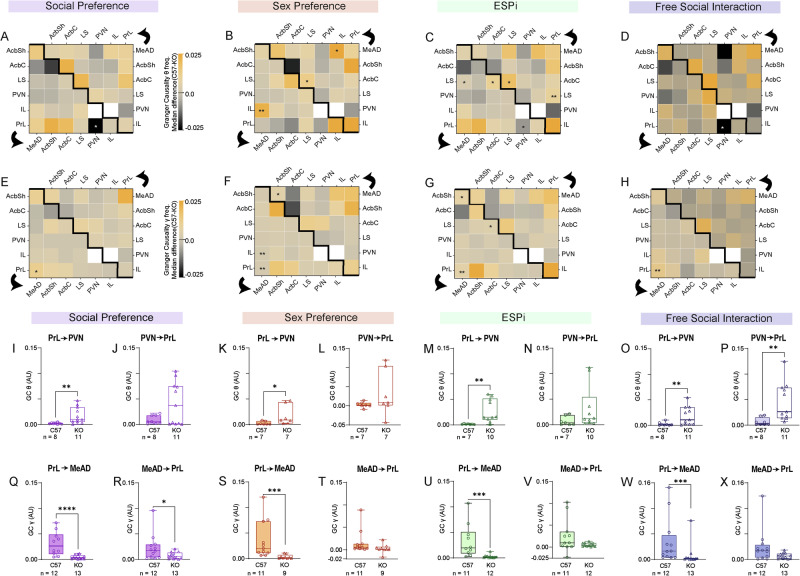
Theta GC values from the PrL to specific areas consistently differ between KO and C57 subjects. **A** Matrix heat-map of the theta band GC differences between C57 and KO mice across all brain region pairs in a SP task session. Note that the lower left matrix represents the GC in one direction, as denoted by the black arrow, while the upper right matrix represents the other direction. White spots represent pairs with inadequate sample size. **B** As in (**A**), for SxP sessions. **C** As in (**A**), for ESPi sessions. **D** As in (**A**), for FSI sessions. **E**–**H** As in (**A**–**D**), for gamma band GC differences. **I** Median theta band GC values from the PrL to the PVN across all SP sessions for C57 (left bar) and KO (right bar) subjects (Mann Whitney test, U = 8, *P* = 0.0018). **J** As in (**I**), for theta band GC values from the PVN to the PrL (U = 34, *P* = 0.4421). **K**–**L** as in (**I**, **J**), for SxP sessions (**M**)**:** U = 5, *P* = 0.0111; (**N**): U = 23, *P* = 0.9). (**M**–**N**) as in (**I**, **J)**, for ESPi sessions (**K**): U = 5, *P* = 0.002; (**L**): U = 18, *P* = 0.1088). (**O**, **P**) As in (**I**, **J**), for FSI sessions (**O**)**:** U = 12, *P* = 0.0068; (**P**): U = 8, *P* = 0.0018). **Q**–**X** As in (**I**–**P**), for gamma GC. **Q**: U = 7, *P* < 0.0001; (**R**): U = 46, *P* = 0.0398; (**S**): U = 4, *P* = 0.0001; (**T**): U = 25, *P* = 0.067; (**U**): U = 33, *P* = 0.0439; (**V**): U = 51, *P* = 0.38; (**W**): U = 15, *P* = 0.0003; (**X**): U = 4, *P* = 0.0678. (**A**–**H**): *p < 0.05, **p < 0.01 FDR corrected Mann Whitney test; (**I**–**X**): *p < 0.05, **p < 0.01, ***p < 0.001, Mann Whitney test.

To further explore the possibility that KO mice exhibit modified synchronization between brain region, we analyzed Granger’s causality (GC), a measure which assesses the predictability of the rhythmicity in one region according to the rhythmicity in the other one, for a given pair of brain regions. The GC was separately calculated for each direction of the possible interaction between each pair of brain regions. Interestingly, the GC level in the PrL to PVN direction in the theta band was consistently higher in KO mice than in C57 animals, with the difference being statistically significant (after correcting for multiple comparisons) across all tasks, other than the SxP task (Fig. 5A–D). In the gamma band, we found consistently and significantly lower GC level in the PrL to MeAD direction for KO mice across all tasks (Fig. 5E–H). Following this screen, we compared GC values between PrL and PVN in each direction across all sessions. We found a significant difference between C57 and KO animals in the theta GC in the PrL to PVN direction in all tasks, while in the other direction (i.e., PVN to PrL), a significant difference was only seen in the FSI task (Fig. 5I–P). Similarly, a significant difference was found in the PrL to MeAD direction for gamma GC values across all tasks, while in the other direction, such a difference was only noted in the SP task (Fig. 5Q–X).

To check if the hyper-synchronization of PrL neural activity with other regions exhibited by KO mice is associated with hyper-activity in this region, we analyzed the multiunit neural activity recorded by us during the same experiments. The mean change (from baseline) in firing rate during interaction, averaged across all brain regions, did not show any significant difference between KO and C57 mice (Fig. S4A–C). As for the PrL, we found no significant difference between the two genotypes in the firing rate change either during the entire 5 min of interaction or during the first minute or during (Fig. S4D–I). Thus, we found no evidence for increased neural activity in KO mice during any of the social discrimination tasks.

Overall, these results suggest that the PrL of KO animals exhibit modified synchronization of neural activity with other regions, such as the PVN and MeAD, during social behavior.

### Optogenetic stimulation of PrL pyramidal neurons abolishes social discrimination

**Fig. 6:**
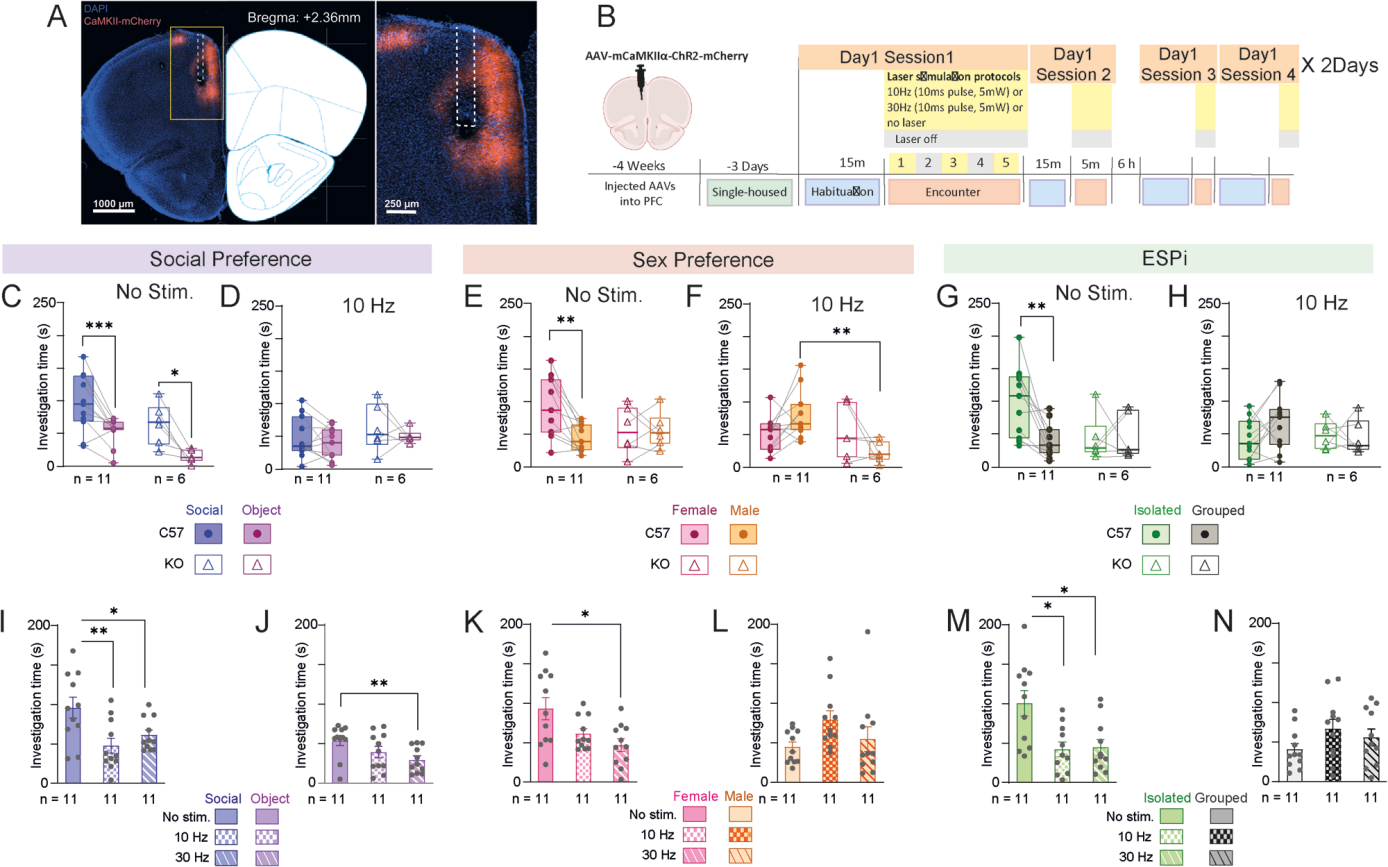
Optogenetic stimulation of PrL pyramidal neurons abolishes any preference for any given stimulus in all social discrimination tasks. **A** A picture showing a coronal brain slice of the prefrontal cortex from a mouse injected with AAV virus carrying Chr2-mCherry under control of the CamK2a promoter on the left, and the corresponding mouse brain atlas slice on the right. The PrL area at higher spatial resolution is shown to the right of the brain atlas profile. **B** The timeline of the various sessions conducted with the virus-injected mice. **C** Median time dedicated by the virus-injected mice implanted with an optic fiber to investigate the animal stimulus (blue) or object (purple) during SP task sessions conducted by C57 (filled circles) and KO (empty triangles) mice without optogenetic stimulation (two-way MM ANOVA. Genotype: F_1, 15_ = 6.793, P = 0.02; Stimulus: F(1,15) = 26.386, P = 0.0001; Interaction: F_1, 15_ = 0.183, P = 0.675). **D** As in (**C**), with a 10 Hz optogenetic stimulation applied throughout the encounter stage (Genotype: F_1, 15_ = 1.421, P = 0.252; Stimulus: F_1, 15_ = 1.425, P = 0.251; Interaction: F_1, 15_ = 0.0.048, P = 0.83). **E**, **F** As in (**C**, **D**), for SxP sessions. **E**: Genotype: F_1, 15_ = 0.705, P = 0.414; Stimulus: F_1, 15_ = 5.547, P = 0.03; Interaction: F_1, 15_ = 5.44, P = 0.034; (**F**): Genotype: F_1, 15_ = 5.72, P = 0.03; Stimulus: F_1, 15_ = 0.000, P = 0.989; Interaction: F_1, 15_ = 6.073, P = 0.026; **G**, **H**. As in (**C**, **D**), for ESPi sessions. **G**: Genotype: F_1, 15_ = 3.312, P = 0.089; Stimulus: F_1, 15_ = 4.513, P = 0.05; Interaction: F_1, 15_ 5.212, P = 0.037; (**H**): Genotype: F_1, 15_ = 0.396, P = 0.539; Stimulus: F_1, 15_ = 0.838, P = 0.374; Interaction: F_1, 15_ = 1.532, P = 0.235. **I** Mean ( ± SEM) time dedicated by the virus-injected mice implanted with an optic fiber to investigate the animal stimulus during SP sessions by C57 subject mice across the three optogenetic stimulation protocols (RM ANOVA, F _2,20_ = 10.369, P = 0.001). **J** As in (**I**), for the object stimulus (F _2,20_ = 8.927, P = 0.002). **K**, **L** As in (**I**, **J**), for SxP sessions (Female: F _2,20_ = 5.836, P = 0.01; Male: F _2,20_ = 2.207, P = 0.136). **M**, **N** As in (**I**, **J**), for the isolated stimulus animal during ESPi sessions (Isolated: F _2,20_ = 8.053, P = 0.003; Grouped: F _2,20_ = 2.356, P = 0.121). **C**–**H** *p < 0.05, **p < 0.01, ***p < 0.001 Šídák’s multiple comparison test after 2-way RM ANOVA; (**I**–**N**) Šídák’s multiple comparison test after RM ANOVA.

The data presented thus far point to the PrL in KO mice as consistently showing hyper-synchronization of LFP theta rhythmicity with other regions during social behavior. We, therefore, hypothesized that the hyper-synchronous theta rhythmicity in the PrL may contribute to the behavioral impairments observed in the KO mice. To further explore this possibility, we examined the effect of stimulating PrL pyramidal neurons in a rhythmic manner during the various discrimination tasks. Accordingly, we used an AAV viral vector to transfect CamK2a-postive neurons in the PrL cortex (presumably pyramidal neurons) of both C57 (*n* = 11) and KO (*n* = 6) animals with Channelrhodopsin-2 (ChR2) (Fig. 6A, B). Fluorescent in situ hybridization analysis revealed that ~90% of the transfected cells were glutamatergic neurons while only ~10% of the transfected neurons were GABAergic (Fig. S5A–D). We then used an optic fiber implanted into the PrL (Fig. S5E) to apply optogenetic stimulation at either 10 or 30 Hz (or applied no stimulation at all) (Fig. S5F, G) during each of the three social discrimination tasks, which were randomized over three days of experimentation (see timeline in Fig. 6B). Notably, When LFP signals were recorded from the various brain regions during 10 Hz optogenetic stimulation, we found a significant increase in mean theta power across all regions, suggesting that this type of stimulation applied to the PrL alone was sufficient to enhance LFP rhythmicity across the whole network (Fig. S6A–C). We found that in all tasks (excluding SP for 30 Hz stimulation), stimulation at 10 and at 30 Hz disrupted the normal preference of C57 mice (Fig. 6C–H and Fig. S6D–F), suggesting that hyper-rhythmic activity of PrL neurons disrupts the subject’s ability to distinguish between social stimuli. Since KO animals performed normally in the SP task despite their hyper-synchronous PrL activity, we examined if further enhancement of rhythmic PrL activity in these animals would impair their SP performance too, similarly to C57 mice. We found that both optogenetic stimulation protocols also disrupted the ability of KO mice to discriminate between the stimulus animal and object during the SP task (Fig. 6C, D and Fig. S6G–I). Altogether, these data suggest that rhythmic stimulation of PrL pyramidal neurons impairs social discrimination in C57 mice, in a manner similar, although not fully identical, to the impaired social discrimination exhibited by KO animals.

Finally, since previous studies suggested that stimulating mPFC pyramidal neurons causes general avoidance of social stimuli and overall reduction in the tendency of a subject to engage in social interaction [25, 42], we examined which behavioral variable was changed upon optogenetic stimulation of PrL pyramidal neurons in C57 mice. We found that in all cases, investigation of the preferred stimulus animal was reduced to the level of the less-preferred one, with the latter level being mostly unaffected (Fig. 6I–N). Since the SxP and ESPi tasks involve discrimination between two stimulus animals, these results suggest that optogenetic stimulation did not cause a general social avoidance, but rather the loss of behavioral preference of one stimulus over the other. Such a specific effect may be caused by a reduction in the valence of the preferred stimulus, which reduces the subject’s motivation to explore the stimulus. However, since the animal stimulus preferred in the SP tasks was the same type as the less-preferred stimulus animals in the other two tasks (i.e., a novel group-housed male mouse), our results contradict the possibility that a change in the absolute valence of the stimulus animal had occurred. Instead, the findings suggest that optogenetic stimulation reduced the relative valence of the preferred stimulus, and hence, the subject’s motivation to explore that stimulus, in the context of two-stimuli choice. To test this idea, we examined the effect of optogenetic stimulation in the case of a one-stimulus choice (stimulus animal vs. an empty chamber). We found that while optogenetic stimulation at 10 Hz abolished the subject’s preference of a social stimulus over an object during a two-stimuli choice (Fig. S6J), it did not impair the tendency of the subject to explore the social stimulus more than an empty chamber (Fig. S6K). We, therefore, concluded that PrL pyramidal neurons may be involved in the display of preference during a competition between two rewarding stimuli, rather than in controlling the absolute motivation to display a specific behavior.

## Discussion

In the present study, we showed that, despite no major difference in their basal sociability, *Cntnap2*-KO animals are impaired in their emotion recognition. Moreover, we found that even WT offspring of *Cntnap2*^+/−^ animals are impaired in this behavior, unlike other social discrimination tasks (such as the SP task) which they perform normally. This result, together with similar results we obtained with *Iqsec2* A350V mice [19] and *Shank3*-KO mice (Jabarin et al., in preparation), suggest that emotion recognition is especially sensitive to ASD-related mutations in mice. This may reflect the subtle behavioral, hormonal and physiological changes presumably distinguishing an emotionally aroused animal from a relaxed animal. The same logic may explain why individuals diagnosed with ASD display specific impairments in emotion recognition, which require them to perceive and interpret subtle motion-induced changes in the facial expressions or body language of others. Thus, the ESP paradigm may be a useful tool for deciphering brain mechanisms underlying ASD-associated atypical social behavior.

Our observation that WT offspring of *Cntnap2*^+/−^ animals were impaired in the ESP behavioral task is in accordance with results of other tasks found with *Neuroligin3*-KO mice, another well-established murine model of ASD [28, 29]. Interestingly, both genes (*Cntanap2* and *Neuroligin3*) encode synaptic proteins and both mutations seems to be associated with a modified excitatory/inhibitory (E/I) balance in the brain [23, 29]. This interesting phenotype may be related to gene-environment interactions, especially as related to the effect of the gut microbiome and its spread from parents to offspring and among littermates, as shown for several mouse models of ASD-associated mutations in synaptic genes [43], including *Cntnap2* [30] (but see also [44]). Regardless of the underlying mechanism, the impaired behavior exhibited by WT offspring of *Cntnap2*^+/−^ parents led us to compare brain activity between KO and C57 mice instead of between KO and WT littermates with the same genetic ground, which is one limitation of our study.

We used multisite electrophysiological recording from behaving mice [31] to explore population neuronal activity in multiple social behavior-associated brain regions during social behavior at the systems level. One intriguing observation was that KO mice exhibited generally stronger theta and gamma rhythms at baseline, even before the beginning of social interactions. This is in accordance with previously published single-cells recordings from the mPFC showing high levels of neuronal activity by *Cntnap2*-KO mice even before stimulus presentation [45]. Given that augmented theta and gamma rhythms are associated with internal states such as arousal and attention [34–38], these results suggest the existence of a high level of a certain internal state in KO mice, which is in accordance with multiple studies demonstrating motor hyper-activity in these mice [22, 23, 30].

Other than the fundamentally higher levels of theta and gamma rhythms, we found a generally high level of theta coherence induced by social encounter in all tasks, suggesting that the various recorded brain regions are over-synchronized in KO mice during social behavior. Such globally high coherence among brain regions may cause behavioral abnormalities by masking social context-induced specific patterns of coherence between brain regions, which may be required for proper recognition of and appropriate responses to specific stimuli [46]. This result is in agreement with a recent study that used fMRI and cFos expression analyses to demonstrate macroscale functional hyper-connectivity in *Cntnap2*-KO mice [47]. Both studies are in accordance with other studies showing that modified synaptic connectivity in the mPFC of *Cntnap2*-KO mice induces modified rhythmic population activity and synchrony in this area [48], as well as altered prefrontal functional connectivity associated with common genetic variants of the humans *CNTNAP2* gene [49]. Together, these studies, which are in agreement with human studies showing a mix of hyper- and hypo-connectivity in ASD individuals [50], support the hypothesis that the behavioral symptoms of ASD are caused by aberrant functional connectivity that may occur as a result of developmental events [51].

We found that the PrL and PVN showed consistent and significantly augmented social behavior-induced hyper-synchrony across all paradigms. These results are very interesting, considering that the PVN is the main source of oxytocin to forebrain areas, in general, and to the mPFC, in particular [52]. Oxytocin is a neuropeptide produced solely in the hypothalamic supraoptic and paraventricular nuclei [53] and is well known for its role in regulating social behavior [54, 55]. Recent studies showed that oxytocin administration can alleviate social behavior deficits exhibited by *Cntnap2*-KO mice [47, 56] and that oxytocin is crucial for murine ESP behavior [18]. Thus, our study complements these earlier efforts by demonstrating modified synchronization between the mPFC and PVN in *Cntnap2*-KO mice during social behavior, thus establishing a functional link between these two regions in the context of emotion recognition in ASD.

Since a previous study showed reduced synchronous activity in PrL pyramidal neurons during emotion recognition task performed by C57BL6/J mice [57], we examined whether the behavioral deficits we observed in KO mice may be caused by hyper-synchronous activity of these neurons. For that, we applied rhythmic optogenetic stimulation to synchronously excite these cells during the various tasks. In agreement with previous studies employing a similar stimulation protocol with all PrL pyramidal neurons [25] or to specific neuronal populations innervating either the NAc [58] or the BLA [42], we found that such stimulation impaired SP in stimulated C57 mice. However, we found that the same manipulation also abolished any preference in the SxP and ESPi tasks. Notably, in all three tasks (SP, SxP, and ESPi), preference abolishment was produced by reducing the time dedicated by the subject to investigate the preferred stimulus animal, whereas no change was observed in the time dedicated to investigating the less-preferred stimulus, be it an object or stimulus animal. This, despite the fact that the same type of stimulus animal served as the preferred stimulus in the SP task and as the less-preferred stimulus in the other two tasks. As such, stimulation-induced reduction in investigation time was neither a general social avoidance, as suggested by previous studies [24, 25], nor stimulus-specific avoidance. Instead, it seems to be a relative valence-specific response [42], expressed as a reduction in the desire to interact with a preferred stimulus more than with other stimuli. This interpretation is supported by our results demonstrating that in the case of a single stimulus in the arena, optogenetic stimulation does not reduce the interest of the stimulated subject in a novel conspecific.

While we observed a significantly higher level of theta coherence across all brain regions and in particular in the PrL of KO animals during social interactions, we did not find evidence for increased neural activity during the same time. This, together with our observation that rhythmic optogenetic stimulation of PrL pyramidal neurons abolished the subjects’ preference in all tasks, supports the idea that hyper-synchronous PrL neural activity, as observed by us in *Cntnap2*-KO mice, may underlie the impaired SxP and ESPi exhibited by these animals. Our observation that these animals function normally in the SP task is most probably explained by the fact that the difference between the two stimuli (object vs. conspecific) is highest in this task, as compared to all other tasks (two conspecifics). This evident difference makes SP the least challenging of all tasks, which may explain its resilient to the naturally-induced hyper-synchronous activity of PrL neurons in *Cntnap2*-KO mice. It should be noted that none of the ASD mouse models examined by us showed impaired SP, while all of them showed impairments in ESP [19].

Overall, our results suggest a pivotal role of the PrL pyramidal neurons in social discrimination, in general, and in emotion recognition, in particular. They also suggest that impaired neural activity of this region, which modifies its synchronization with other social behavior-associated brain regions, such as the PVN and MeAD, is involved in the deficits exhibited by *Cntnap2*-KO mice in terms of emotion recognition. Such impairments in PrL activity may also underlie similar deficits observed in other murine models of ASD [19], suggesting a common brain pathway that integrates the effects of multiple ASD-associated mutations in synaptic proteins into a specific deficit in emotion recognition.

## Methods

### Animals

#### Maintenance

Adult male and female C57BL/6J mice (C57, 12-14 weeks old) were purchased from Envigo (Rehovot, Israel). *Cntnap2*^−/−^ mice [22] (KO; 12–14 weeks old) used in this study were maintained by crossing *Cntnap2*^+/−^ (heterozygous) mutant males with C57BL/6J females. All KO and wild type (WT) mice used in this study were obtained by crossing heterozygous animals and born with the expected Mendelian frequencies. For generating pure WT mice, WT males and females were crossed to produce pure WT offspring that were later used for behavioral testing at eight weeks of age. All animals were kept in sex-matched groups of 2–5 mice per cage at the animal facility of the University of Haifa under veterinary supervision, in a 12 h light/12 h dark cycle (lights on at 19:00), with *ad libitum* access to food (standard chow diet, Envigo RMS, Israel) and water. Experiments were performed in the dark phase of the dark/light cycle in a sound- and electromagnetic noise-attenuated chamber. After surgery, electrode array-implanted mice were singly housed so as to not disturb the electrode array.

#### Genotyping

Ear tissue samples were collected from offspring mice aged 21 days for genotyping by polymerase chain reaction (PCR) using the following primers:

WT forward primer: TCAGAGTTGATACCCGAGCGCC;

WT reverse primer: TGCTGCTGCCAGCCCAGGAACTGG;

Mutant forward primer: TTGGGTGGAGAGGCTATTCGGCTATG;

Mutant reverse primer: TGCTGCTGCCAGCCCAGGAACTGG.

#### Ethical statement

All experiments were performed according to the National Institutes of Health guide for the care and use of laboratory animals and approved by the Institutional Animal Care and Use Committee (IACUC) of the University of Haifa (Ethical approval #1077U).

### Behavioral assays

Male and female KO, WT, and pure WT mice performed multiple social discrimination tasks, as previously described [19, 26, 59]. Each session comprised 15 min of habituation in the arena with empty triangular chambers in opposing corners of the arena, as previously described [27], followed by the subject mice performing the task of interest for 5 min (encounter).

In the SP task, subjects were exposed to an adult novel group-housed male mouse (social) and a Lego toy (object), separately located in individual chambers at opposing corners of the arena. In the SxP task, subject mice encountered novel adult male and female stimulus animals. In the ESPs task, subject mice encountered a novel stimulus animal stressed by 15 min restraint in a 50 ml Falcon tube with an opening for air, and a non-stressed (naive) stimulus animal. In the ESPi task, subject mice encountered novel group-housed and socially isolated (for 1–2 weeks) stimulus animals. Each isolated stimulus animal was used for two non-consecutive tests. All stimulus animals used in all four tests were novel adult C57BL/6 J mice. In the FSI task, subject mice freely interacted with a group-housed, same-sex age-matched novel stimulus animal for 5 min.

### Electrophysiology

#### Electrode array surgery

Electrode arrays (EArs) were implanted as previously described [31]. Briefly, mice were anesthetized using isoflurane (induction 3%, 0.5–0.8% maintenance in 200 mL/min air; SomnoSuite) and placed over a custom-made heating pad (37°C) in a standard stereotaxic device (Kopf Instruments, Tujunga, CA). Two burr holes were drilled to allow placement of ground and reference wires (silver wire, 127 µm, 300–500 Ω; A-M Systems, Carlsborg, WA). Two watch screws (0-80, 1/16”, M1.4) were inserted into the temporal bone to support the electrode array with dental cement. Four points (at coordinates: AP = 2 mm, ML = −0.3 mm; AP = 1 mm, ML = −2.3 mm; AP = −2 mm, ML = −2.3 mm; AP = −2 mm, ML = −0.3 mm) were marked over the left hemisphere with a marker. The skull covering these marked coordinates was removed after smoothening the bone with a dental drill, and the exposed brain was kept moist with cold, sterile saline. We custom-designed the EAr from 8 to 12 individual 50 µm formvar-insulated tungsten wires (50-150 kΩ, #CFW2032882; California Wire Company) to target the PrL, IL, AcbC, AcbSh, LS, PVN and MeAD. Before implantation, the EAr was dipped in 1,1′-Dioctadecyl-3,3,3’,3’-tetramethylindocarbocyanine perchlorate (Dil, 42364, Sigma-Aldrich) to visualize electrode locations post-mortem. The reference and ground wires were inserted into their respective burr holes and the EAr was lowered onto the surface of the exposed brain using a motorized manipulator (MP200; Sutter Instruments). The dorsoventral coordinates were estimated using the depth of the electrode targeting the PVN (AP = −1 mm, ML = −0.3 mm), which was lowered slowly to −4.7 mm. The EAr and exposed skull with the screws were secured with dental cement (Enamel plus, Micerium). Mice were sub-cutaneously injected with Baytril (5 mg/kg; Bayer) and Norocarp (5 mg/kg; Carprofen, Norbrook Lab) post-surgery and allowed to recover for three days. After surgery, implanted mice (subjects) were singly housed so as to not disturb the electrode array.

#### Electrophysiological and video recording setup

Following a brief exposure to isoflurane, subjects were attached to a headstage board (RHD 16 ch, #C3334, Intan Technologies) through a custom-made Omnetics-to-Mill-Max adaptor (Mill-Max model 852-10-100-10-001000). Behavior was video recorded from above the arena using a monochromatic camera (30 Hz, Flea3 USB3, Flir). Electrophysiological recordings were made with the RHD2000 evaluation system using an ultra-thin SPI interface cable connected to the headstage board through a manual commutator (Dragonfly Research and Development, Inc. Model # FL-89-OPT-12-C). In the case of combining electrophysiology and optogenetics, the optic fiber and SPI cable were fed through a motorized commutator (AlphaComm-1, Alpha Omega, Israel) to reduce cable entanglement during the tasks. Electrophysiological recordings (sampled at 20 kHz) were aligned with recorded video using a TTL trigger pulse and by recording camera frame strobes.

### Electrophysiological recordings

We recorded the behavior and neural activity of 15 KO and 11 C57 adult male mice (Table S1) while targeting distinct brain regions, as described above. We discounted electrodes mistargeted into the dorsomedial hypothalamic nuclei (DMD) and amygdalo-hippocampal region (AhiAL) from further analysis as these regions do not present specific association to social discrimination behavior. Before experiments, the mice were briefly exposed to isoflurane, and the EAr was connected to the evaluation system. Each recording session was divided into two 5-min stages, namely, a baseline period during which time the subject was alone in the arena in the presence of two empty chambers (or no chambers, in the case of the FSI task) and a task (encounter) period when the subject performed the task in the presence of stimuli. Each subject was evaluated over three sessions of each task. The subjects performed the SP and FSI tasks with 10-min intervals separating each of the sessions. The ESPi and SxP tasks were next performed with similar intervals. Four sessions spread 6 h apart were recorded daily, two in the morning and two in the afternoon, (see timeline in Fig. S2A). Sessions were excluded from further evaluation only when the headstage detached from the EAr or in case of a missing video recording from a session. This accounts for the unequal number of sessions and subjects across tasks.

### Optogenetic experiments

#### Surgery

For the optogenetic stimulation experiments, we performed surgeries on C57 (*n* = 11) and KO male mice (*n* = 6). These mice were anesthetized using isoflurane (induction 3%, 0.5–0.8% maintenance in 200 mL/min of air; SomnoSuite) and placed under a heating pad attached to stereotaxic apparatus, in a manner similar to the EAr surgeries described above. The mice were then injected with 300 nl of a viral vector encoding an excitatory opsin (ssAAV-1/2- mCaMKIIα-hChR2(H134R)mCherry-WPRE-SV40p(A), produced by Viral Vector core, ETH, Zurich) with a titer of 2.9*10^12^ viral genomes per ml in the left-hemisphere prefrontal cortex (PFC, AP: 1.9 mm, ML: 0.4 mm, DV: 2.2 mm). An optic fiber (200 um, 0.66 NA, flat bottom, 3 mm long, Doric lenses) was implanted into the PFC (AP: 1.9 mm, ML: 0.4 mm, DV: 2 mm), with the exposed skull along with part of the ferrule holding the optic fiber were stuck together using dental cement (Enamel plus, Micerium). In the case of combining optogenetic stimulation with electrophysiology, KO mice were implanted with EAr, that included an optic fiber glued to the electrode targeting PFC. Along with PFC, the EAr had electrode targeting AcbC, AcbSh, LS, PVN and MeAD. The mice were held in social isolation for recovery three days post-surgery, and then kept for four weeks in group-housing conditions to allow for full expression of the opsin. To mimic the experimental conditions of EAr-implanted mice, mice with optic fiber implants were kept in isolation for three days before the experiments and throughout this period.

#### Behavioral experiments with optogenetic stimulation

Mice (C57 and KO) undergoing optogenetic stimulation were video-recorded while performing a similar battery of tasks as EAr-implanted mice. Each subject underwent three sessions of the SP, ESPi, and SxP tasks, each with a different stimulation protocol, with the order of tests and stimulation protocols being randomized. The stimulation protocols were: [1] no stimulation throughout the encounter period; [2] stimulation at 10 Hz (1 min long, 473 nm, 5 mW, 10 ms pulse) three times, with a 1-min inter-stimulation interval; and [3] similar stimulation as the second protocol, but at 30 Hz. Before the experiments, the mice were lightly anesthetized with isoflurane and connected to the laser (473 nm, model FTEC2471-M75YY0, Blue Sky Research) for light delivery, manually controlled using a pulse stimulator (Master8, AMPI). The mice were then allowed to habituate to the arena and empty chambers for 15 min, after which time actual experiments with stimuli in chambers were recorded for 5 min.

### Combined electrophysiology and optogenetics experiments

The KO mice were subjected to same optogenetic stimulation paradigm while recording behavioral experiments as C57. In addition, the neural activity was recorded from KO brains as previously mentioned in electrophysiology recording section, while stimulation or no stimulation with blue laser.

### Histology

Subjects were trans-cardially perfused, and their brains were kept cold in 4% paraformaldehyde in PBS for 48 h. Brains were washed carefully in PBS, sectioned (50 µm) in the horizontal plane with a vibrating blade microtome (VT 1200 s, Leica) and collected onto microscope slides. Electrode marks were visualized (Dil coated, Red) against DAPI-stained sections with an epifluorescence microscope (Ti2 eclipse, Nikon). The marks were used to locate the respective brain regions based on the mouse atlas [60]. In optogenetic stimulation task subjects, mCherry expression and fiber optic location in PFC slides were validated under the epifluorescence microscope using coronal brain slices.

### Data analysis

#### Behavioral analysis

##### Social discrimination (SP, SxP, ESPs and ESPi tasks)

Subject behavior was tracked using the TrackRodent software, as previously described [26, 27, 61].

#### FSI task

##### Markerless pose estimation

DeepLabCut software (v.2.3.5, maDLC) [32] was used to track the positions of the subject and stimulus during FSI sessions. The training set included 600 frames from 3 sessions out of a total of 58 sessions (C57 and KO mice). The following body parts were marked for both subject and stimulus in each frame: Ear_left, Ear_right, Nose, Neck, Trunk, Lateral_right, Lateral_left, Tail_base and Tail_mid (Fig. 2J). The model was trained by 2*106 iterations with default parameters (training frames selected by k-means clustering of each of the three videos, trained on 95% of labeled frames, initialized with dlcrnet_ms5, batch size of 8). The model, when tested on the remaining 5% of labeled frames, gave a training error of 3.32 pixels and a test error of 5.43 pixels. Further average root mean square error was less than 4 pixels for all body parts, except for the subject’s nose, which was less visible due to top-view video recordings and electrical cable tether, marked in the labeled frames (Fig. S2B–E).

##### Supervised classification of subject behavior

To identify behaviors across the entire video dataset, we trained supervised random forest classifiers (RF) using a subset of data (5 sessions of 58) from maDLC-analyzed sessions using SimBA’s GUI. Briefly, the sessions were smoothed with a Stravinsky-Golay filter of 100 ms, and outliers were corrected using SimBA’s default criterion (1 AU, median of the data). During free interaction sessions, behaviors of interest included *Sniff_StimBody, Sniff_Stim_Anogenital, Being_sniffed, NosetoNose, Aggressive, Moving, Sit_Idle* and *Grooming*. In detail, each of these behaviors was defined as follows:

*Sniff_StimBody:* The subject sniffs the body of the stimulus spanning the whole body, excluding the head and anogenital regions.

*Sniff_Stim_Anogenital:* The subject sniffs the anogenital part of the stimulus body, including any area below the trunk.

*Being_Sniffed:* The stimulus sniffs any part of the subject body, excluding the head.

*NosetoNose:* The subject and the stimulus sniff each other’s facial/head regions.

*Aggressive:* The subject shows aggressive behavior directed towards the stimulus, like biting, tail rattling, trying to mount the stimulus, and holding the stimulus tail with hind paws.

*Moving:* The subject is moving around in the arena, irrespective of the presence of stimulus.

*Sit_Idle:* The subject sits in a non-motile state, irrespective of the stimulus location in the arena.

*Grooming:* The subject grooms or scratches regions of its body and face with either forepaws or hind paws.

An independent RF classifier was trained to classify each of these behaviors. The parameters and the complete RF used to train these classifiers are shared as experimental data. Moreover, classification reports from sklearn.metrics.classification_report were used to quantify and plot precision, recall, and f1 values for each RF classifier. Those behaviors with an RF model that had a f1 value above 0.8 (*Sniff_StimBody, Sniff_Stim_Anogenital, Sit_Idle*, and *Grooming*, Fig. S2B–E) were included for further comparisons between WT and KO subjects. The remaining 53 sessions were analyzed for the RF classifiers of the four behaviors and further analyzed for electrophysiological parameters, similar to the TrackRodent-analyzed data of the SP, ESPs, ESPi and SxP tasks.

#### Electrophysiological data analysis

##### LFP power

Only brain regions recorded for more than five sessions across at least three mice in both KO and C57 mice were analyzed. All signals were analyzed using custom-made codes written in MATLAB 2020a. We excluded signals recorded during 30 saround stimulus removal and insertion times to avoid any artifacts due to these actions. The signals were first down-sampled to 5 kHz and low-pass filtered to 300 Hz using a Butterworth filter. The power and time for the different frequencies were estimated using the “spectrogram” function in MATLAB with the following parameters: Discrete prolate spheroidal sequences (dpss) window = 2 s; overlap = 50%; increments = 0.5 Hz; and time bins = 0.5 s. The power of each frequency band (theta: 4–12 Hz and gamma: 30–80 Hz) was averaged for both the baseline and encounter periods (5 min each). Changes in theta (ΔθP) and gamma (ΔγP) power for each brain region were defined as the mean difference in power between the encounter and baseline periods.

##### Coherence

We used the “mscohere” function of MATLAB to estimate coherence values using Welch’s overlapped averaged periodogram method. The magnitude-squared coherence between two signals, x, and y, was defined as follows:

$${{Coherence}}_{{xy}}=\frac{{S}_{{xy}}}{\sqrt{{S}_{{xx}}\,{S}_{{yy}}}}$$

where ${S}_{{xy}}$ is the cross-power spectral density of x and y$,\,{S}_{{xx}}$ is the power spectral density of x and$\,{S}_{{yy}}$ is the power spectral density of y. All coherence analysis was quantified between brain region pairs involved in at least five sessions of behavior tasks. Coherence for the baseline period was quantified as the average coherence of all brain region pairs for each task. Changes in coherence (ΔθCo and ΔγCo) during the encounter period between a pair of brain regions were calculated as follows:

$${Change\; in\; Coherence}=\frac{{{{\rm{\mu }}}}({{Coherence}}_{{encounter}}-{{Coherence}}_{{baseline}})}{{{{\rm{\sigma }}}}({{Coherence}}_{{encounter}}-{{Coherence}}_{{baseline}})}$$

where, Coherence_encounter_ is the absolute coherence value between a pair of regions within a frequency band during a whole encounter period. Coherence_baseline_ is the absolute coherence value between a pair of regions within a frequency band during an entire encounter period.

##### Granger causality

We employed the multi-variate GC toolbox [62] to calculate GC values separately for baseline and encounter periods between brain regions for each task and rhythm. For GC analysis, LFP signals were measured at a reduced sampling rate of 500 Hz.

We used the “tsdata_to_infocrit” function to determine the model order of the vector autoregressive (VAR) model. The median model order for all three tasks was 38 (Bayesian information criterion). To further fit the VAR model to our multi-session, multivariate LFP data, the “tsdata_to_var” function of LWR (Levinson-Whittle recursion) in the regression mode, and a median model order of 38 was used separately for the baseline and encounter periods of each task. Next, we estimated the autocovariance sequence of the fitted VAR model with the “var_to_autocov” function. To maximize the computational efficiency of the function, an acmaxlags of 1500 was chosen. This process did not violate the autocovariance VAR model, as was estimated by the “var_info” function. Finally, we calculated the pairwise conditional frequency-domain multivariate GC matrix using the “autocov_to_spwcgc” function and summed the GC for the relevant frequency band (theta or gamma) using the “smvgc_to_mvgc” function.

### Statistical analysis

Statistical analysis was performed using GraphPad Prism 10 or SPSS 24. Normal distribution of the data was tested using Shapiro-Wilk tests. A paired t-test or Wilcoxon matched-pairs signed rank test was performed to compare various stimuli or conditions for the same group, while an unpaired t-test or Mann-Whitney test was used to compare variables between distinct groups. ANOVA was applied to the dataset when comparing a parameter among multiple groups and *post hoc* multiple comparison tests were performed with Šídák’s correction if a main effect was observed. Similarly, when comparing a parameter that had repeated measurements among multiple groups, repeated measures (RM) ANOVA was performed on the dataset and, in case of a main effect, post hoc comparisons between the groups was adjusted using Šídák’s corrections. In the case of GC, multiple Mann-Whitney test results were corrected using FDR correction. When comparing two factors and the interaction between these factors among multiple groups, two-way ANOVA was used, followed with Šídák’s correction of multiple comparisons if a main effect was observed. Similarly, when one of the factors was repeated measurement of the two factors and interaction between them was compared between multiple groups, RM two-way ANOVA was performed. When there was significant main effect, Šídák’s correction of multiple comparisons was performed in post hoc tests. All parameters and results of all statistical tests are detailed in Table S2.

## Supplementary information

Supplementary figures legends

Figure S1. C57BL/6J and Pure WT female mice do not exhibit a preference to any of the stimuli in the ESPi task

Figure S2. Timeline and SimBA details

Figure S3. Differences between C57 and KO mice in change in LFP power along the time course of the various tasks

Figure S4. No differences between C57 and KO mice in change in firing rate during the encounter stage of the various tasks

Figure S5. Optogenetic stimulation details

Fig. S6. Results of optogenetic stimulation

Supplementary Table 1: Consolidated data of each figure used for statistical tests

Supplementary Table 2: Summary of statistical tests

## Data Availability

All the data generated from experiments and the custom codes used to evaluate the conclusions of this paper are uploaded to 10.5281/zenodo.12954413.
