# Supplementary material for: Impaired emotion recognition in Cntnap2-deficient mice is associated with hyper-synchronous prefrontal cortex neuronal activity
Source: Mol Psychiatry. 2024 Sep 17;30(4):1440–52. doi: 10.1038/s41380-024-02754-8 (PMC11919685; doi:10.1038/s41380-024-02754-8)
Supplement: Supplementary file 1 — Supplementary figures legends [file 41380_2024_2754_MOESM1_ESM.docx]

**Supplementary figures**

**Figure S1. C57BL/6J and Pure WT female mice do not exhibit a preference to any of the stimuli in the ESPi task.**

1. The breeding scheme of C57BL/6J female mice.
2. Median (in box plot^) time dedicated by C57BL/6J female mice to investigating the isolated (green) stimulus or the grouped (grey) stimulus during the 5 min encounter period of the ESPi task (paired t-test, n = 20 sessions, t_20_ = 1.429, p = 0.1658).
3. As in **A**, for the pure WT female mice.
4. As in **B**, using pure WT female mice (t_19_ = 0.836, p = 0.4141).
5. Relative discrimination index (RDI) for C57BL/6J (One sample t-test, t_29_ = 0.715, p>0.05) and pure WT (t_18_ = -0.531, p>0.05) female mice.

^Box plot represents 25 to 75 percentiles of the distribution, while the bold line is the median of the distribution. Whiskers represent the smallest and largest values in the distribution.

*p<0.05, **p<0.01, ****p<0.0001, One sample or Paired samples t-test.

**Figure S2. Timeline and SimBA details**

1. Timeline of the various tasks conducted by the electrode array-implanted mice.
2. Heat-map of classification metrics from SimBA for presence or absence of *Sniff_StimBody* events in all the frames in which the Random forest model was validated. A snapshot of the SimBA-annotated behavior event is added to the right of the heat-map of the metrics.
3. As in **B**. for *Sniff_Stim_Anogenital* events.
4. As in **B**. for *Sit_Idle* events.
5. As in **B**. for *Moving* events.
6. Fluorescence image of the horizontal section at -4.7 mm from Bregma of a subject’s left-hemisphere with electrode-tip locations marked by DiI (red) and gross anatomy depicted by DAPI-stained nuclei (blue). The corresponding panel from the atlas is attached to the right side of the image. Scale = 1 mm.
7. Median θ power (dB/Hz) of the baseline period from the first session for each subject (Unpaired t test, n = 7 brain regions, t_12_ = 4.281, p = 0.0004).
8. As in **L**. median γ power (t_12_ = 2.395, p = 0.0338).

*p<0.05, **p<0.01, ****p<0.0001, Unpaired t-test.

**Figure S3. Differences between C57 and KO mice in change in LFP power along the time course of the various tasks**

1. Mean (±SEM) change (from the average during baseline) in LFP theta power (∆θ power) averaged across all brain region, along the 5-min baseline stage and 5-min encounter stage of the SP task, compared between C57 (circles) and KO (triangles) mice. Time 0 represents stimuli introduction to the arena (Two-way MM ANOVA; Time: F _1.9,22.84_ = 76.883, p<0.0001, Genotype: F _1,12_ = 11.328, p= 0.006, Interaction: F _1.9,22.84_ = 49.244, p<0.0001).
2. As in **A**, for the SxP task (Time: F_1.55,18.62_ = 51.377, p<0.0001, Genotype: F _1,12_ = 14.42, p = 0.003, Interaction: F _1.55,18.62_ = 1.07, p = 0.347).
3. As in **A**, for the ESPi task (Time: F 1_.84,22.08_ = 118.07, p<0.0001, Genotype: F _1,12_ = 4.23, p = 0.423, Interaction: F _1.66,23.3_ = 21.43, p = 0.003).
4. As in **A**, for the FSI task (Time: F _1.66,23.3_=84.33, p<0.0001, Genotype: F _1,12_=0.039, p= 0.847, Interaction: F _1.66,23.3_ = 21.43, p<0.0001).

**E-H.** As in **A-D**, for the change in LFP gamma power (∆γ power) (SP- Time: F_1.8,21.64_ = 98, p<0.0001, F_1,12_ = 2.324, p = 0.153, Interaction: F_1.8,21.64_ = 26.7, p<0.0001; SxP- Time: F_1.9,22.98_ = 91.55, p<0.0001,Genotype: F_1,12_ = 7.269, p = 0.019, Interaction: F_1.9,23.98_ = 1.098, p = 0.348; ESPi- Time: F_1.9,23.9_ = 178.29, p<0.0001, Genotype: F_1,12_ = 1.083, p = 0.319, Interaction: F_1.9,23.9_ = 1.0262, p = 0.001; FSI- Time: F_1.8,21.97_ = 60.3, p<0.0001,Genotype: F_1,12_ = 0.506, p = 0.497, Interaction: F_1.8,2197_ = 0.599, p = 0.544).

*p<0.05, **p<0.01, ****p<0.0001, Unpaired t-test with Sidak correction.

**Figure S4. No differences between C57 and KO mice in change in firing rate during the encounter stage of the various tasks**

1. Median change (from the average during baseline) in firing rate averaged across all brain region, for the entire 5-min encounter stage of the SP task, compared between C57 (circles) and KO (triangles) mice. Time 0 represents stimuli introduction to the arena (Mann-Whitney Test, U = 9, p = 0.053).
2. As in **A**, for the SxP task.
3. As in **A**, for the ESPi task.
4. As in **A**, specifically for the PrL.
5. As in **D**, just for the first minute of the encounter stage.

**F-G.** As in **D-E**, for the SxP task.

**H-I.** As in **D-E**, for the ESPi task.

**Figure S5. Optogenetic stimulation details**

1. A picture by a fluorescent microscope of a brain slice from the mPFC of a stimulated animal, showing the tract of the implanted optic fiber on the background of: I) a DAPI staining; II) mChery expression in cells infected with CamK2a-Chr2-mCherry virus, all of which are marked by white circles; III). Slc in situ hybridization prode, labeling glutamatergic neurons, with ChR+/Slc+ cells labeled by red circles and ChR=/Slc- cells labeled with yellow circles; IV) Merge if II and III (no labeled cells).
2. A pie chart showing the percentage of Slc+ cells from Chr+ cells, analyzed across 6 slices from 5 animals.

**C-D.** As in **A-B**, using GAD probe to label GABAergic neurons (7 Slices from 5 animals).

1. Coronal brain section panels of the left hemisphere from the mouse atlas at Bregma +2.46, +1.96 and +1.54 with depth of optic fiber marked for C57 (X) and KO (●) subjects.
2. Spectrograms of recorded mPFC LFP signals for three protocols of optogenetic stimulation in C57 mice expressing ChR2.0 in the left mPFC along with the implanted electrode and optic fiber. The left image corresponds to spectrogram reflecting 1 min of no stimulation from an electrode placed in the mPFC. The middle image is of a 10 Hz (473 nm, 5 mW, 10 ms pulse) stimulation and the right image is of a 30 Hz stimulation. Note the harmonics seen at multiples of the stimulation rate.
3. Power-spectral density plots of LFP signals from the mPFC electrode shown in B, for no stimulation, and for 10 Hz and 30 Hz stimulation.

**Fig. S6. Results of optogenetic stimulation**

1. Median change in theta power recorded from 6 KO animals implanted with both microelectrode array and optic fiber in the PrL cortex infected with CamK2a-ChR2_mCherry AAV viruses, averaged across all recorded brain areas without (left bar) and with (right bar) 10 Hz optogenetic stimulation given during the SP task (Paired t-test, t_5_ = -3.772, p = 0.013).
2. As in A, for the SxP task (t_5_ = -3.734, p = 0.014).
3. As in A, for the ESPi task (t_5_ = -7.94, p = 0<0.001).
4. Median time dedicated by the virus-injected mice implanted with an optic fiber to investigate the animal stimulus (blue) or object (purple) during SP task sessions conducted by C57 (filled circles) and KO (empty triangles) mice, with a 30 Hz optogenetic stimulation (2 way MM ANOVA, SP- Time: F_1,15_ = 5.534, p = 0.033, Genotype: F_1,15_ = 0.472, p = 0.503, Interaction: F_1,15_ = 2.53, p = 0.133).
5. As in D, for the SxP task.
6. As in D, for the ESPi task.
7. Relative discrimination index (RDI) for C57 (filled bars) and KO (empty bars) mice in all three optogenetic stimulation conditions in the SP task. (One sample t-test, KO 10Hz: t_5_ = 4.546, p = 0.006; WT no stimulation: t_10_ = 3.557, p = 0.005; WT 30Hz: t_10_ = 4.694, p = 0.001).
8. As in **A** for the SxP task (WT no stimulation: t_10_ = 4.006, p = 0.002; WT 10Hz: t_10_ = -2.4, p = 0.037).
9. As in **A** for the ESPi task (WT no stimulation: t_10_ = 4.863, p = 0.001).
10. A scheme of the SP task.
11. A scheme of the one-stimulus task.
12. Median time dedicated by C57 mice (n = 5) implanted with an optic fiber to investigate the animal stimulus (blue) or object (purple) during SP task sessions without (left bars) and with (right bars) 10 Hz optogenetic stimulation (Paired t-test, t_4_ = 2.924, p = 0.043, 10Hz: t_4_ = 1.428, p = 0.227.
13. As in L, for the same animals conducting the one-stimulus task, with only social stimulus in the arena, with 10 Hz optogenetic stimulation (t_4_ = 4.112, p = 0.015).

**p<0.01, ***p<0.001, One sample t-test or Paired t-test

**Supplementary Table 1: Consolidated data of each figure used for statistical tests**

The dataset comprises of separate sheets for each figure panel’s data used for statistical analysis.

**Supplementary Table 2: Summary of statistical tests**

The dataset includes worksheets of statistical tests results for each figure panel.
