# Supplementary figures and images for: Impaired emotion recognition in Cntnap2-deficient mice is associated with hyper-synchronous prefrontal cortex neuronal activity
Source: Mol Psychiatry. 2024 Sep 17;30(4):1440–52. doi: 10.1038/s41380-024-02754-8 (PMC11919685; doi:10.1038/s41380-024-02754-8)

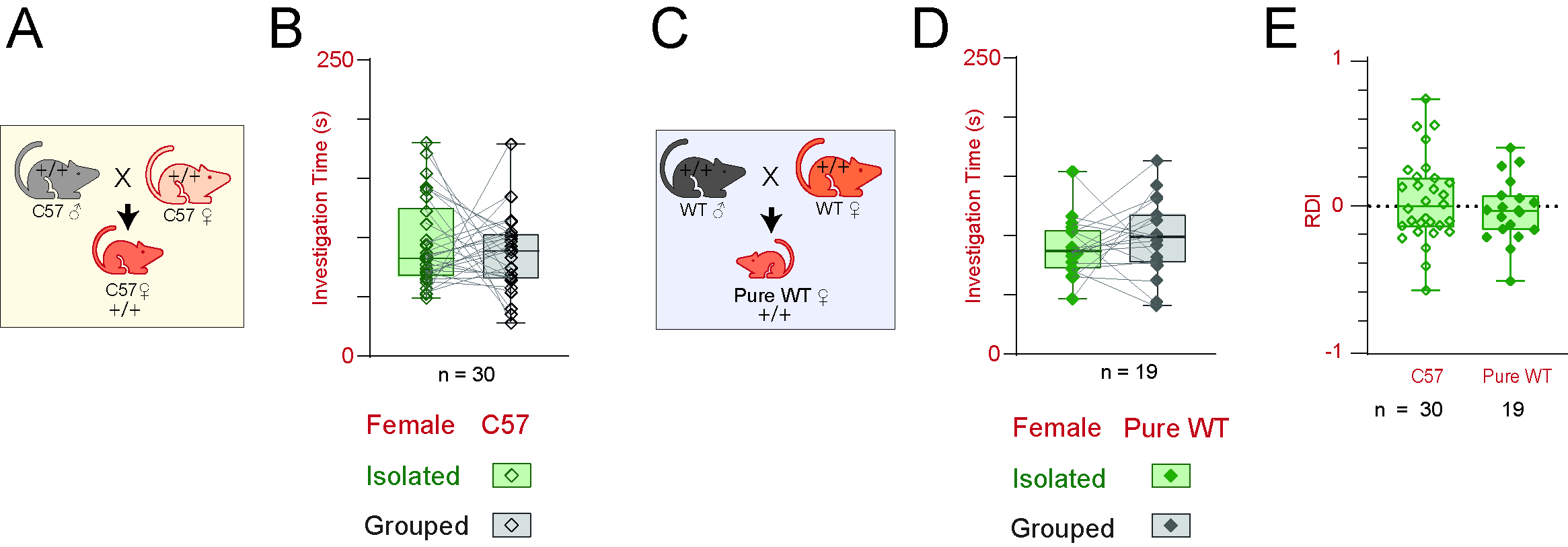

Supplement: Supplementary file 2 — Figure S1. C57BL/6J and Pure WT female mice do not exhibit a preference to any of the stimuli in the ESPi task [file 41380_2024_2754_MOESM2_ESM.tif]

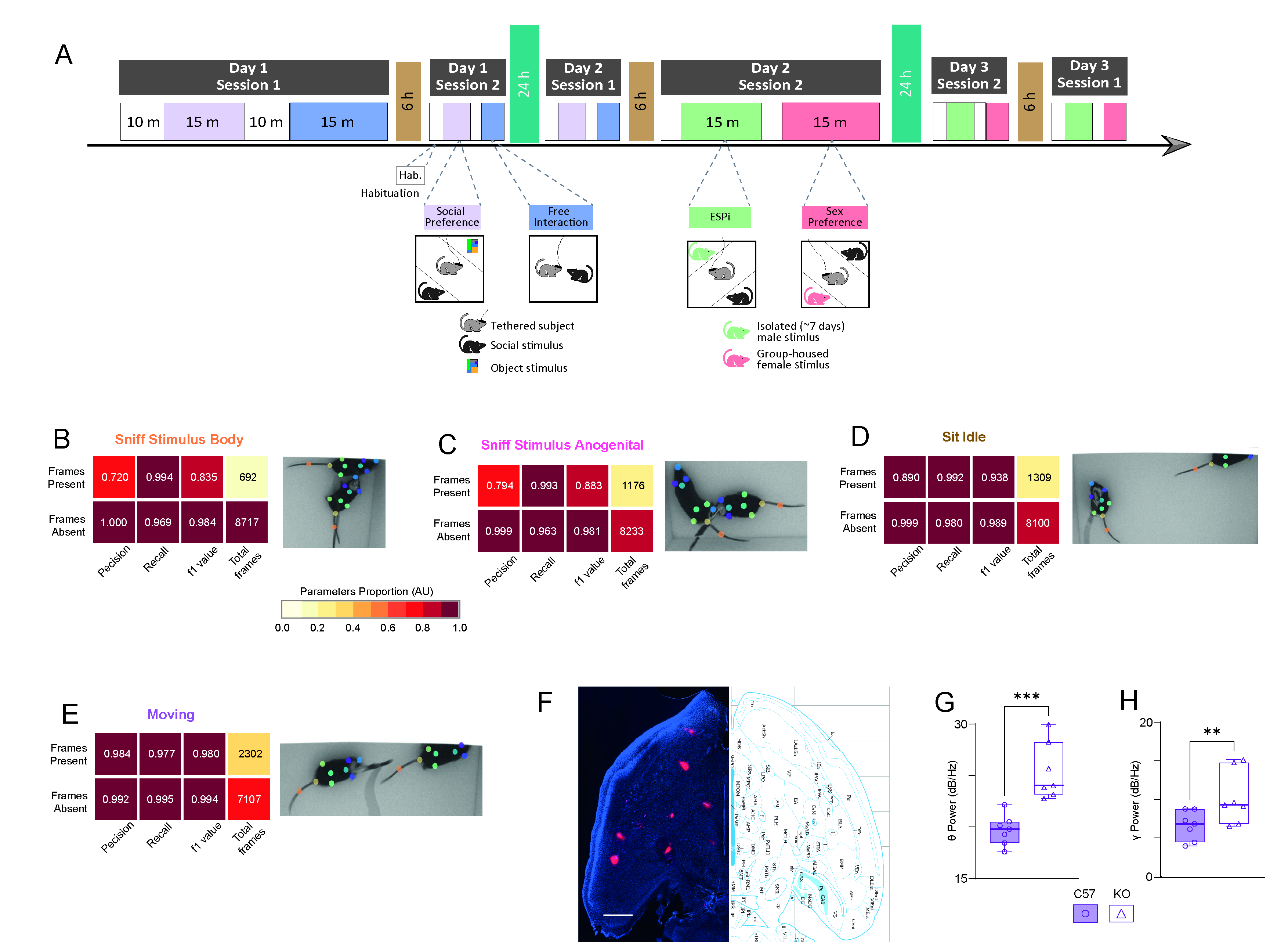

Supplement: Supplementary file 3 — Figure S2. Timeline and SimBA details [file 41380_2024_2754_MOESM3_ESM.tif]

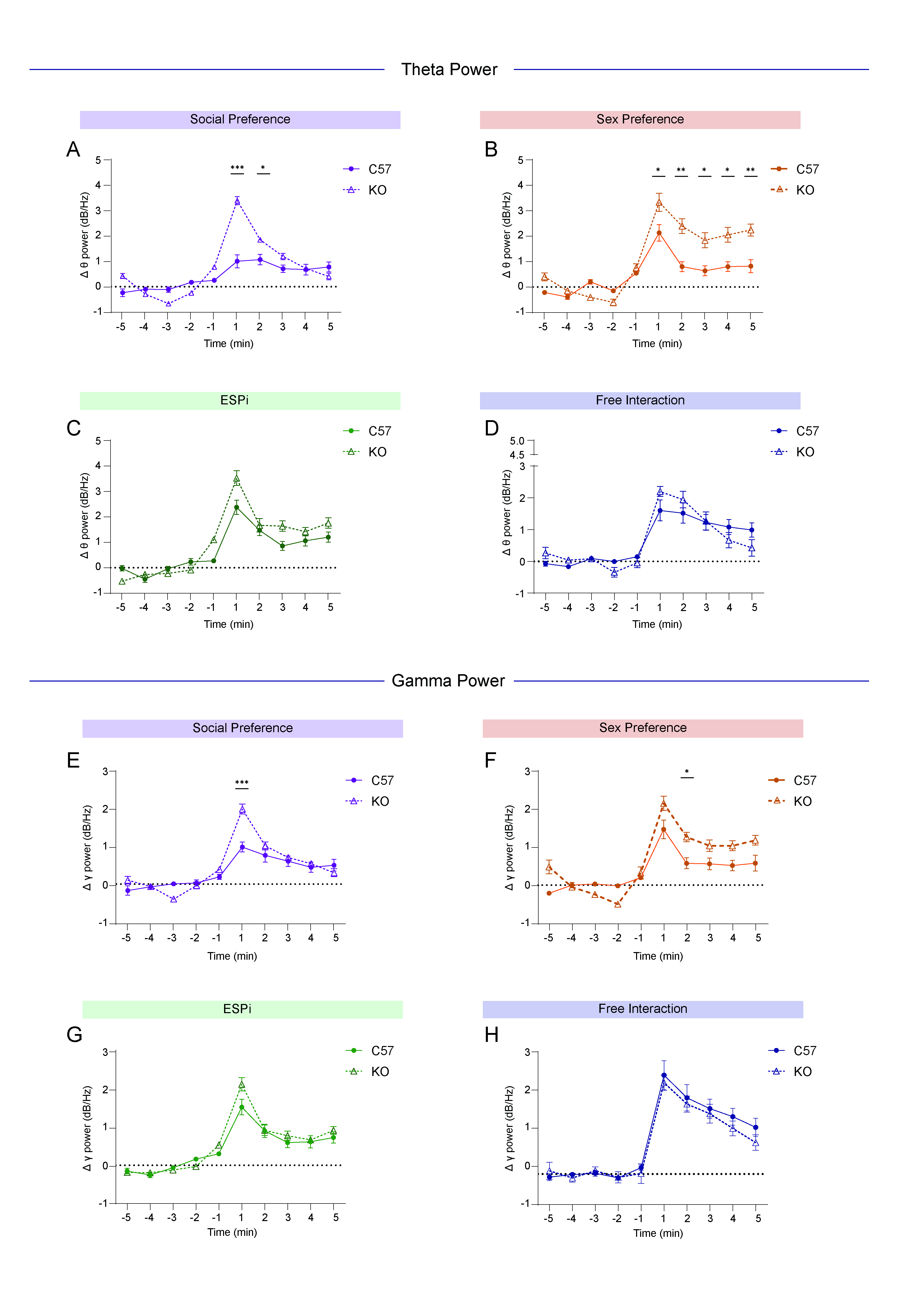

Supplement: Supplementary file 4 — Figure S3. Differences between C57 and KO mice in change in LFP power along the time course of the various tasks [file 41380_2024_2754_MOESM4_ESM.tif]

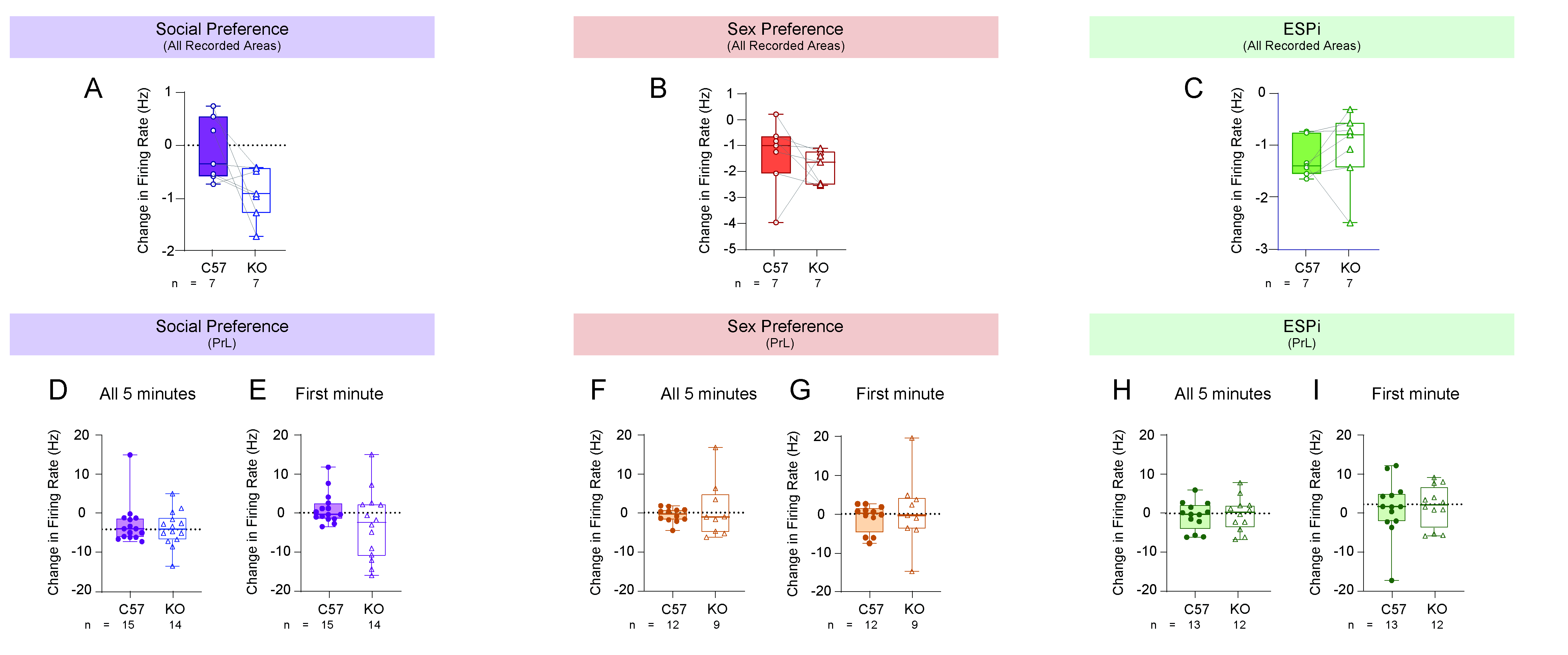

Supplement: Supplementary file 5 — Figure S4. No differences between C57 and KO mice in change in firing rate during the encounter stage of the various tasks [file 41380_2024_2754_MOESM5_ESM.tif]

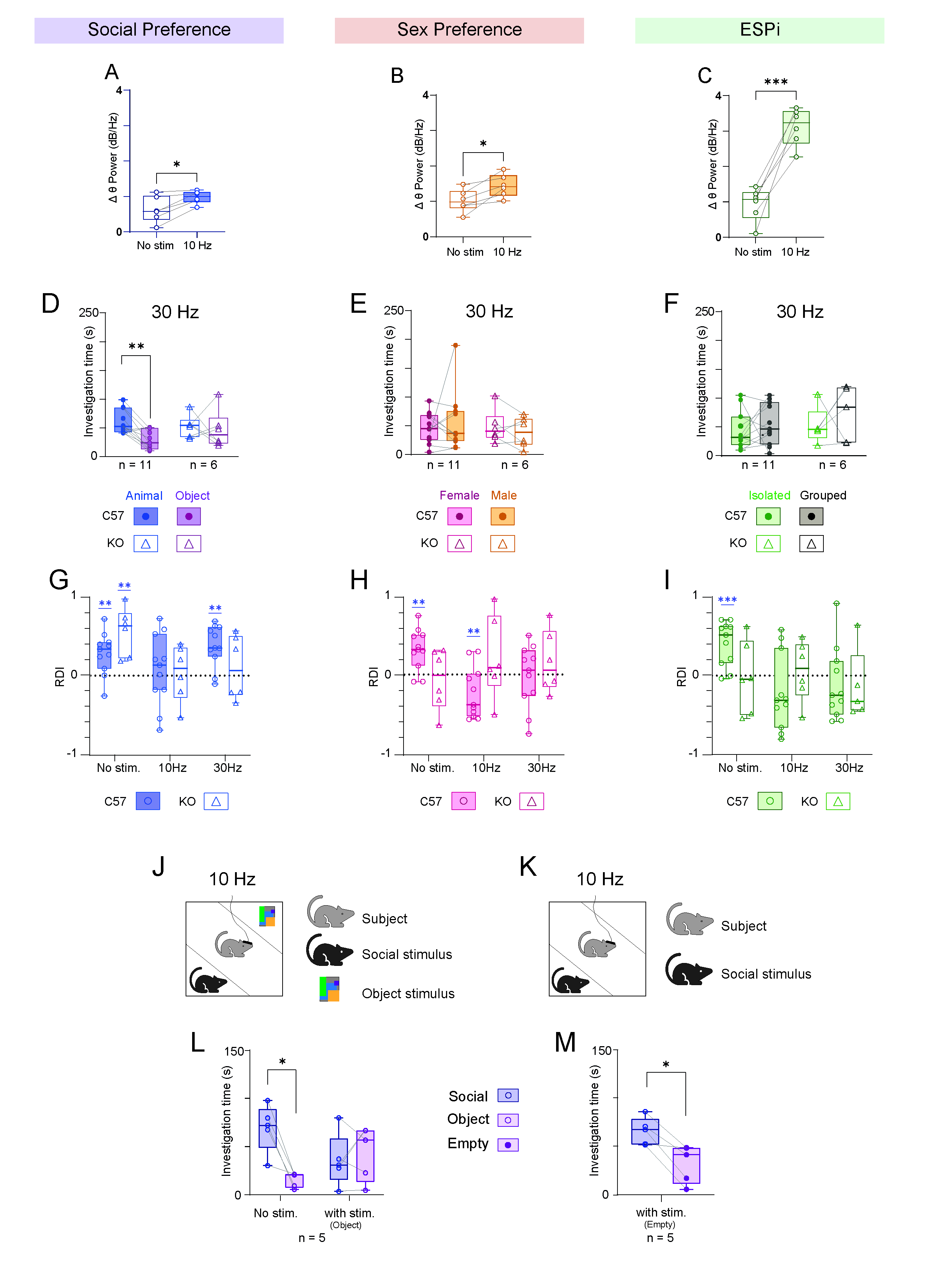

Supplement: Supplementary file 7 — Fig. S6. Results of optogenetic stimulation [file 41380_2024_2754_MOESM7_ESM.tif]
